# A Case of Pulmonary Nodular Lymphoid Hyperplasia Responding to Corticosteroid Treatment

**DOI:** 10.7759/cureus.37870

**Published:** 2023-04-20

**Authors:** Jonathan Teow Koon Goh, Issam Al Jajeh, Jessica Han Ying Tan

**Affiliations:** 1 Respiratory Medicine, Sengkang General Hospital, Singapore, SGP; 2 Pathology, Sengkang General Hospital, Singapore, SGP

**Keywords:** hemoptysis, chronic cough, interstitial lung disease, pulmonary lymphoproliferative disease, pulmonary nodules, pulmonary nodular lymphoid hyperplasia

## Abstract

Pulmonary nodular lymphoid hyperplasia (PNLH) is a rare non-neoplastic disease that presents with mass lesions in the lung. It is radiologically difficult to differentiate it from adenocarcinoma of the lung or pulmonary lymphoma. There has been no consensus regarding the treatment of PNLH; however, in many case series, patients usually undergo surgical resection for diagnostic and therapeutic purposes. Here, we present the case of a 60-year-old Chinese male who presented with cough and hemoptysis. A computed tomography scan of the thorax revealed a mass-like lesion. A biopsy was performed which showed lymphocytic pneumonitis. He was treated with a tapering dose of corticosteroids with good clinical and radiological outcomes. Upon a subsequent review of the case, a diagnosis of PNLH was made. This case report suggests that corticosteroids may be an alternative therapy to surgical resection. They have the advantage of being non-invasive and can be used in patients who are otherwise not surgical candidates due to other comorbidities. However, further research is required before we can recommend corticosteroids as a treatment for PNLH.

## Introduction

Pulmonary nodular lymphoid hyperplasia (PNLH) is a rare non-neoplastic lymphoid hyperplasia that presents with mass lesions in the lung. Its existence has been controversial, and it was initially termed pseudolymphoma by Saltzstein in 1963 [1]. The term nodular lymphoid hyperplasia was coined by Kradin and Mark in 1983 [2] and was included in the World Health Organization (WHO) classification of lung tumors in 2001 [3].

While there has been no consensus regarding the treatment for PNLH, it is difficult to differentiate it from lung adenocarcinoma; hence, most patients undergo surgical resection for diagnostic and therapeutic purposes [4,5]. Here, we present a case of PNLH which fully resolved after a course of corticosteroid (prednisolone) treatment.

## Case presentation

**Figure 1:**
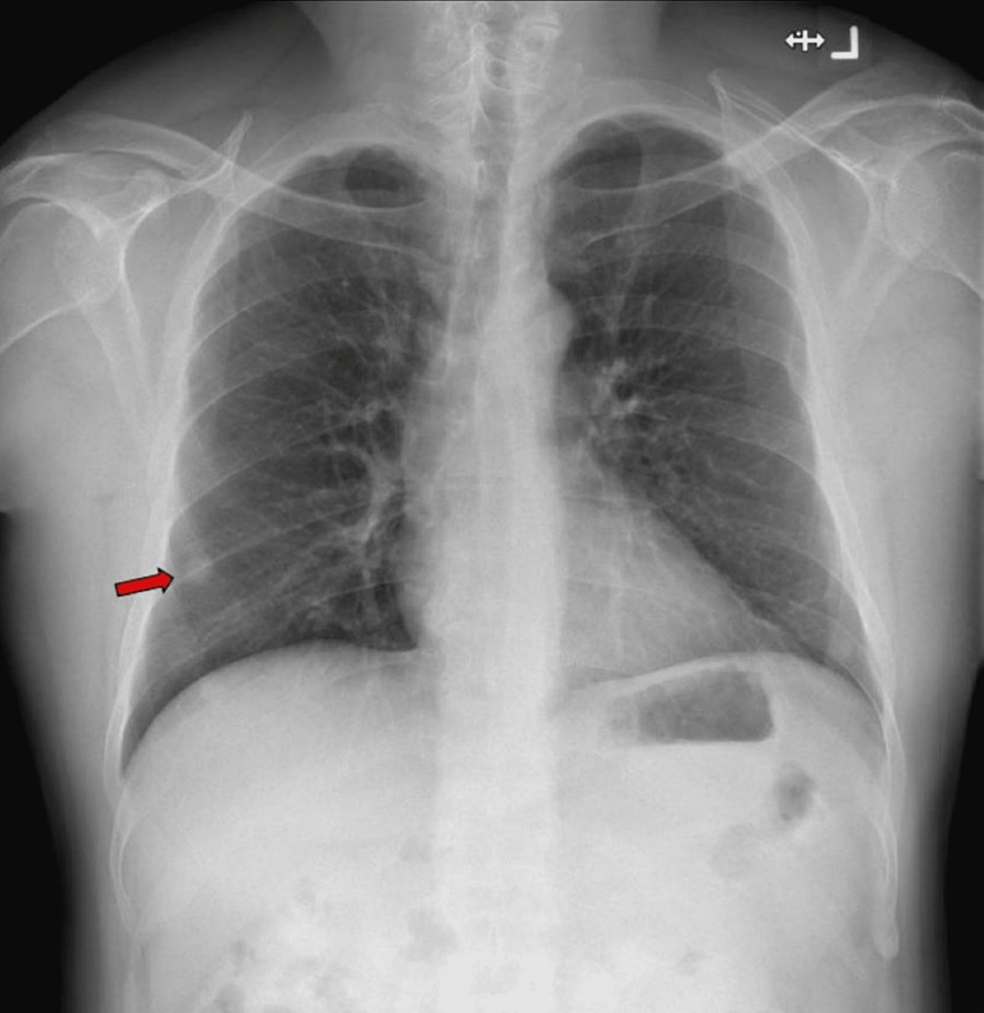
Chest radiograph on presentation to the emergency department showing a well-rounded opacity in the right middle zone.

**Figure 2:**
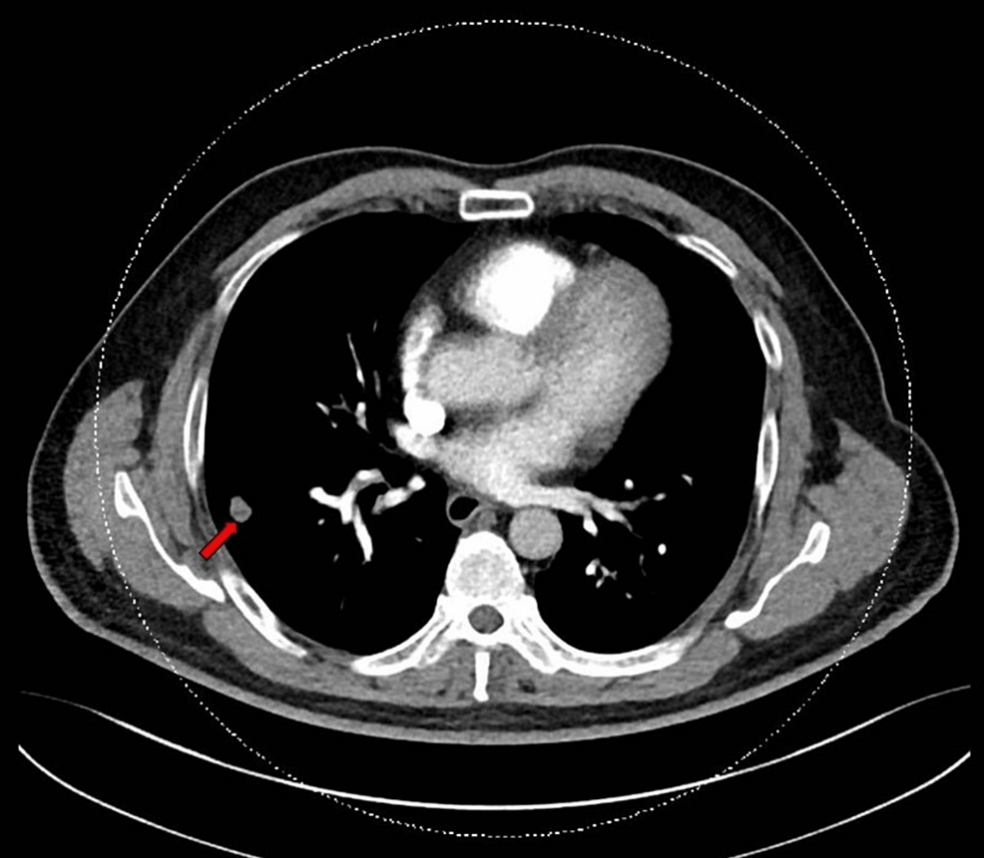
Computed tomography of the thorax showing the mildly lobulated right nodule.

A 60-year-old Chinese male, a non-smoker, presented to the emergency department following a road traffic accident in September 2019. He had no known history of any autoimmune or connective tissue diseases. A computed tomography (CT) scan of the thorax was performed as a chest radiograph showed a right lower zone well-circumscribed opacity. The CT of the thorax revealed a right lower lobe superior segment mildly lobulated nodule measuring 1.3 cm. A biopsy via either surgical resection or a CT-guided biopsy was offered; however, the patient opted to follow up with interval CT of the thorax. Subsequent CT of the thorax in August 2020 and September 2021 showed that the nodule was stable (Figures 1, 2).

**Figure 3:**
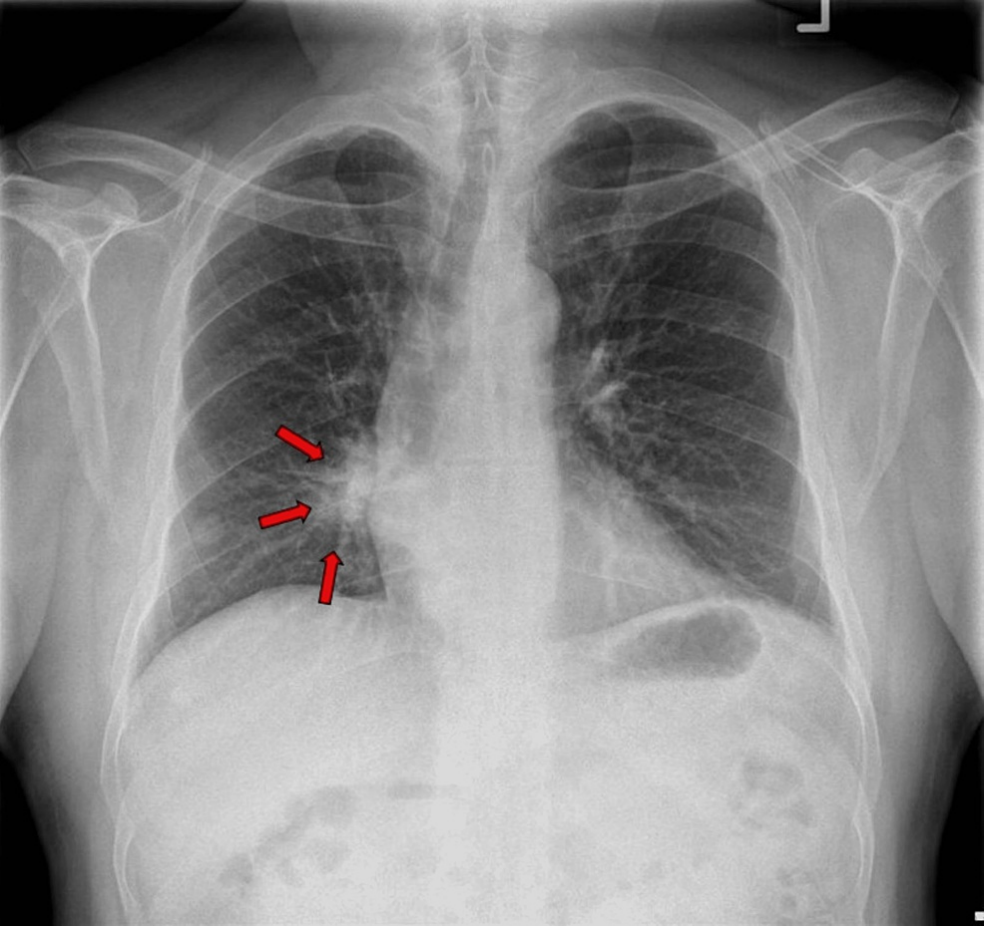
Chest radiograph after the patient complained of a cough and hemoptysis showing new right hilar mass-like consolidation.

**Figure 4:**
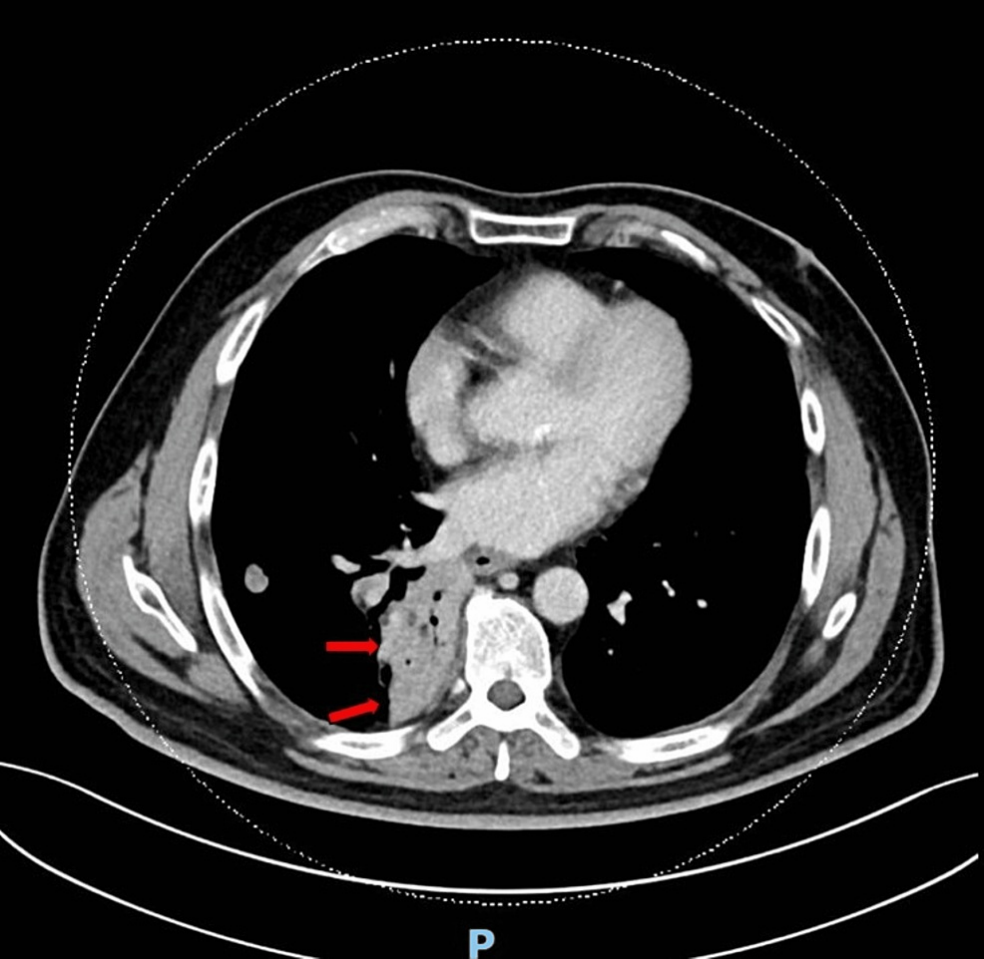
Computed tomography of the thorax axial view with new mass-like consolidation in the right lower lobe.

**Figure 5:**
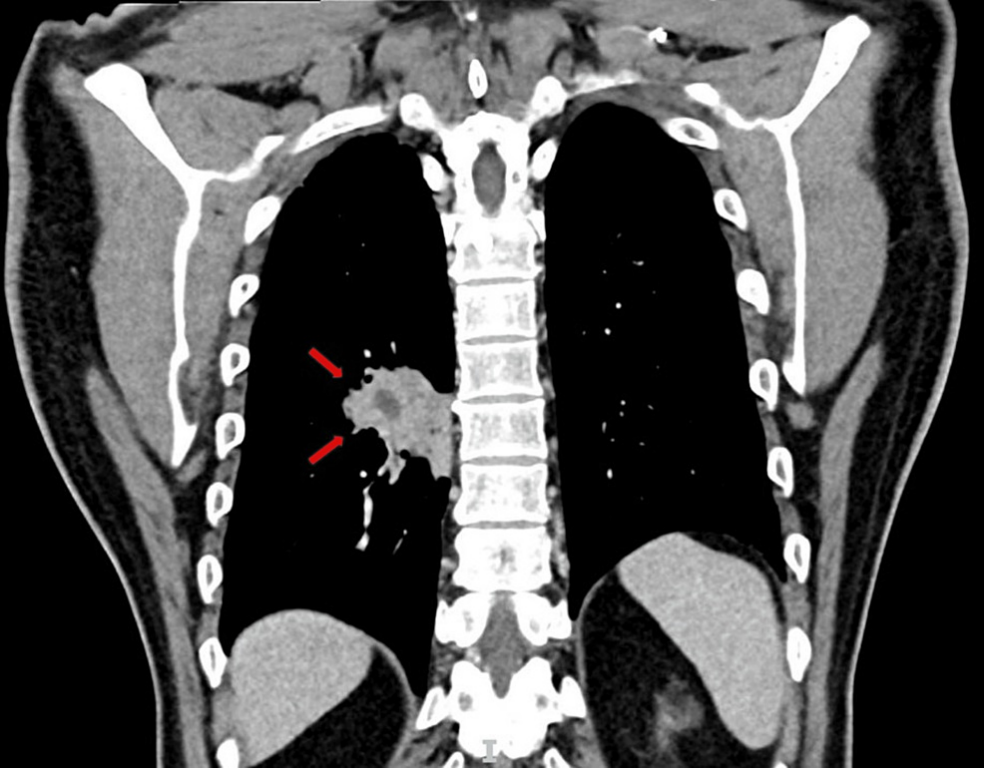
Computed tomography of the thorax coronal view with new mass-like consolidation in the right lower lobe.

In April 2022, the patient complained of a cough with small amounts of hemoptysis for the past two months. This was associated with a weight loss of 5 kg over the same period. Detailed history and examination did not reveal any signs or symptoms of autoimmune or connective tissue diseases. A chest radiograph was performed in April 2022 which showed a right hilar mass that was new compared to the previous radiographs. A CT of the thorax, abdomen, and pelvis was performed in May 2022 which showed that while the initial nodule was stable, there was a new mass-like consolidation in the medial right lower lobe measuring 4.4 cm × 7.2 cm. A bronchoscopy with bronchoalveolar lavage (BAL) and transbronchial lung biopsy (TBLB) under fluoroscopy of the right lower lobe was performed. No endobronchial lesions were seen, and neither was there any blood or active bleeding. BAL microbiology was negative including for acid-fast bacilli (AFB) smear and culture. BAL cytology showed inflammatory exudates which were mainly neutrophils, while TBLB histology showed bronchial tissue with lymphocytic and plasma cell-rich infiltrates with no malignancy detected. Immunohistochemistry of the lymphocytic infiltrates was inconclusive for light chain restriction due to limited lesional tissue (Figures 3-5).

**Figure 6:**
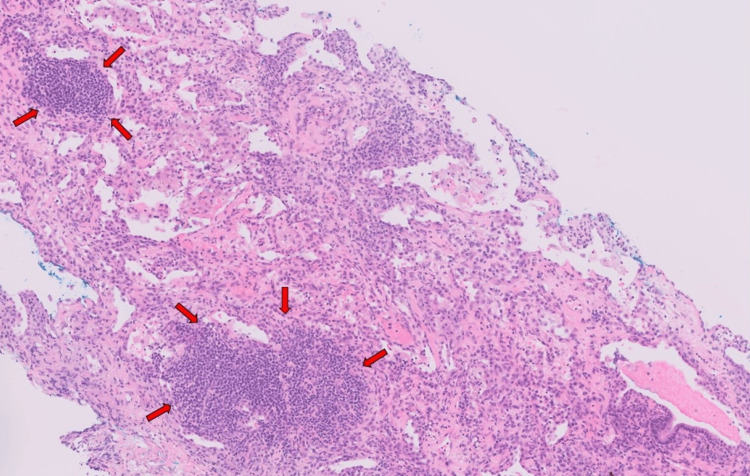
Histology of the right lower lobe mass-like consolidation obtained by CT-guided biopsy. Hematoxylin and eosin stain at ×20 magnification showing centrilobular lymphoid aggregates.

**Figure 7:**
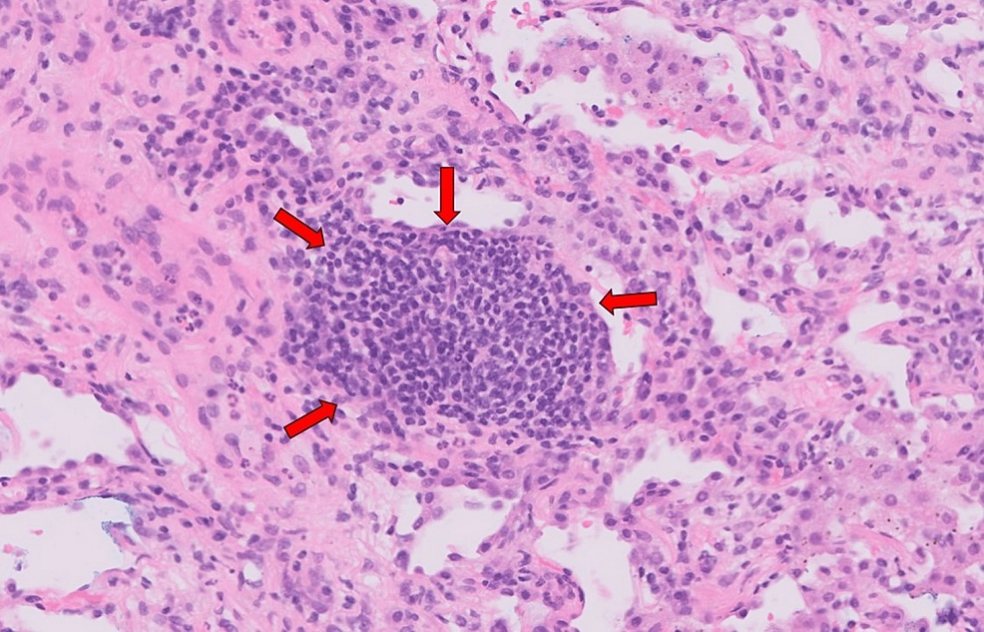
Histology of the right lower lobe mass-like consolidation obtained by CT-guided biopsy. Hematoxylin and eosin stain at ×40 magnification showing centrilobular lymphoid aggregate with reactive pneumonitis inflammatory pattern in lobules.

A repeat chest radiograph in June 2022 showed that the hilar nodular consolidation was still persistent; hence, a CT-guided biopsy of the right lower lobe mass-like consolidation was performed in June 2022. Histology showed a micronodular lymphocytic pneumonitis pattern which involved centrilobular areas with extension into small septa and peri-septal alveolar walls. It was associated with mild ongoing alveolar cell injury, inflammation, and repair. There was no evidence of malignancy of carcinoma or lymphoma. There were no granulomas, giant histiocytes, notable capillaritis or capillary thrombi, necrotizing vasculitis, or viral cytopathy identified. The proximal acinar interstitium was diffusely expanded by mild-to-moderate lymphoplasmacytic infiltrates with evenly spaced small lymphoid aggregates and occasional follicles. The immunohistochemistry profile was consistent with reactive infiltrates. No follicular colonization, lymphoepithelial lesions, or light chain restriction was identified (Figures 6, 7).

The decision was made to treat this as lymphoid bronchiolitis. An autoimmune screen was performed which included antinuclear antibody, extractable nuclear antigen antibody panel, extended myositis panel, rheumatoid factor, and anti-cyclic citrullinated peptide antibody which were all negative. The erythrocyte sedimentation rate was raised at 86 mm/hour, and C-reactive protein was 8.1 mg/L.

**Figure 8:**
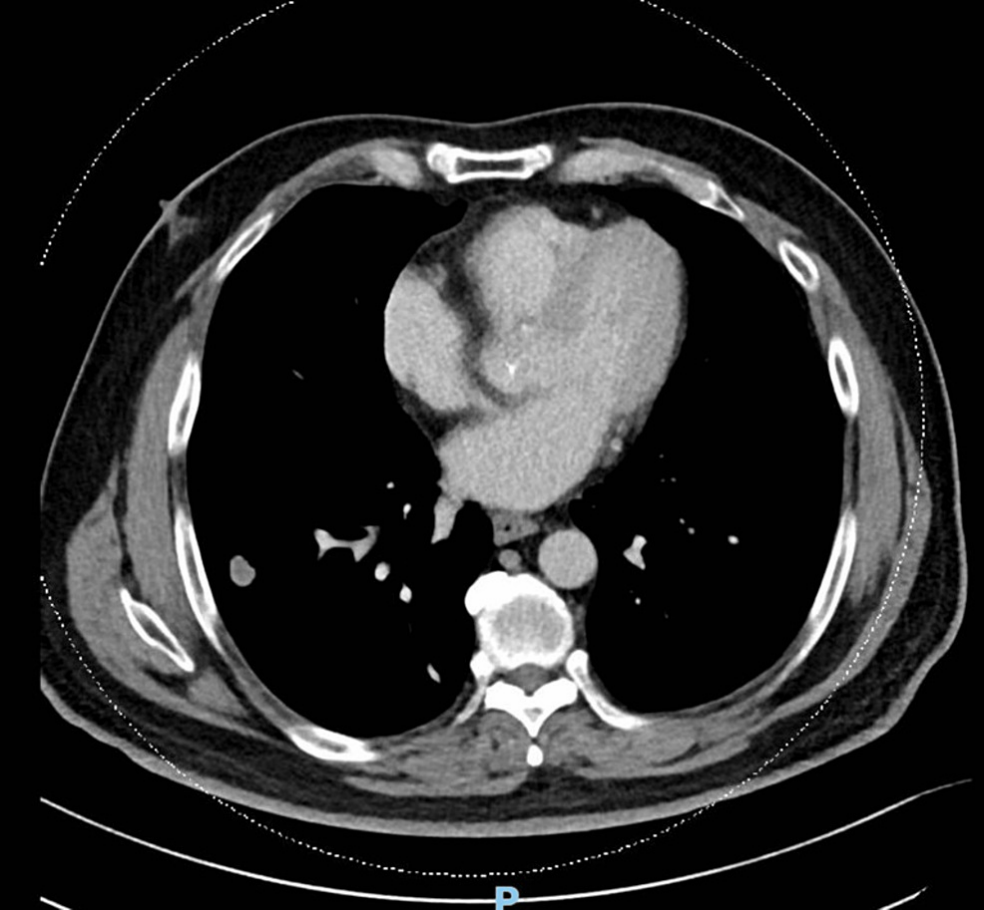
Computed tomography of the thorax showing resolution of the mass-like consolidation after corticosteroid treatment. The original lung nodule remains unchanged.

**Figure 9:**
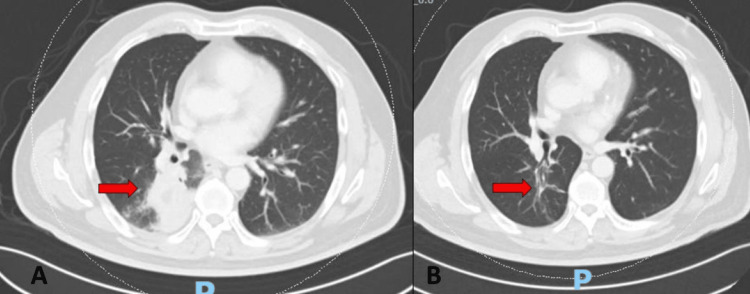
Computed tomography of the thorax view of the same location before (A) and after (B) corticosteroid treatment.

The patient was initiated on oral prednisolone 40 mg OM with a tapering regime over 12 weeks. Within three weeks of initiating steroids, the patient reported that his cough was significantly improved, and hemoptysis had resolved. Repeat chest radiograph at the three-week mark showed interval improvement of the right hilar mass-like consolidation. CT of the thorax was repeated in November 2022 after the completion of steroids which showed a marked improvement of the solid mass-like consolidation in the superior segment of the right lower lobe with residual scarring and peribronchial thickening. The initial lobulated nodule remained stable. The patient reported being asymptomatic with no further episodes of weight loss or hemoptysis. He did not suffer from any complications of prolonged corticosteroid use. He continues to remain on follow-up with our service for interval surveillance of the original lung nodule (Figures 8, 9).

## Discussion

PNLH is a rare benign disorder and our understanding of it is limited. There does not seem to be any sex predilection and patients are mostly middle-aged (median age of 57 years with an age range of 25 to 72 years) [4], although there are reports of patients as young as 12 years old [6].

Patients are usually asymptomatic (53.7%) [4] or may present with cough and/or hemoptysis [4,7]. While there have been reports of associations with Sjogren’s disease [8], there are no clear risk factors for PNLH [4,9]. Case series have identified patients with previous tuberculosis who subsequently developed PNLH, although no clear association could be drawn from such small numbers [4]. Regardless, our patient had no known prior history of tuberculosis and the BAL fluid AFB smear and culture were negative.

On CT scan, PNLH usually presents as a single mass-like lesion, occasionally two or three lesions, with no predilection to any lobe. PNLH rarely presents with multiple bilateral lesions [4,7,9]. Occasionally, PNLH may present with regional lymphadenopathy [4,9].

PNLH demonstrates histological and immunohistochemical features of a benign process with reactive germinal center formation. These include follicular hyperplasia and interfollicular polyclonal plasmacytosis with varying degrees of fibrosis [7,9]. Immunoglobulin G (IgG) 4 (IgG4) plasmacytosis has been reported in some cases. Guinee et al. reported a significantly raised number of IgG4-positive plasma cells in PNLH, with an increased IgG4:IgG ratio, raising the concern of IgG4-related disease causing PNLH [10]; however, subsequent studies did not show the ratio to be elevated [6,7].

Although there is no consensus regarding treatment, PNLH is difficult to distinguish clinically from malignancy, especially lung adenocarcinoma and pulmonary lymphoma; hence, many patients undergo diagnostic and curative surgical resection with good outcomes [4,9]. Although there are case reports that PNLH may resolve spontaneously [11-13], conversely, Park et al. reported a case that worsened radiologically over a period of two years [14].

To our knowledge, only two other case reports utilized corticosteroids to treat PNLH. Moriyama et al. used a starting oral prednisolone dose of 0.5 mg/kg/day for a duration of seven months, while Judge et al. used a starting oral prednisolone dose of 40 mg daily tapered over six months. Although both used dosing regiments that were similar to ours, we tapered our regiment over a shorter period of three months. Both Moriyama et al. and Judge et al. reported resolution of symptoms after the initiation of corticosteroids; however, Moriyama et al. did not report any improvement in radiology, while Judge et al. reported radiological stability or improvement [5,15].

In our patient, the use of corticosteroids resulted in not only the resolution of symptoms but also radiological resolution. The success of prednisolone in achieving clinical and radiological resolution suggests that this could be a less invasive alternative to surgical resection. Furthermore, not all patients are surgical candidates for lung resection. We do recognize that prolonged corticosteroid use can lead to potentially serious side effects; hence, we should consider its use only when patients are symptomatic or show radiological worsening.

## Conclusions

PNLH is a rare condition that remains poorly understood. Although there is no consensus regarding treatment, in most cases, patients undergo diagnostic and therapeutic resection as it is difficult to distinguish PNLH radiologically from lung adenocarcinoma or lymphoma.

In this case, we treated a case of PNLH with a course of corticosteroids with good clinical and radiological resolution. This case serves to suggest that there could be a non-invasive alternative to surgical resection in treating PNLH. Further research is needed before we can recommend this as a potential treatment for symptomatic patients.
